# Phenolic Content Analysis of Two Species Belonging to the *Lamiaceae* Family: Antioxidant, Anticholinergic, and Antibacterial Activities

**DOI:** 10.3390/molecules29020480

**Published:** 2024-01-18

**Authors:** Ashwell R. Ndhlala, Mesut Işık, Arzu Kavaz Yüksel, Emrah Dikici

**Affiliations:** 1 Green Biotechnologies Research Centre, School of Agricultural and Environmental Sciences, University of Limpopo, Private Bag X1106, Sovenga 0727, South Africa; ashwell.ndhlala@ul.ac.za; 2 Department of Bioengineering, Faculty of Engineering, Bilecik Şeyh Edebali University, Bilecik 11230, Turkey; 3 Department of Food Technology, Technical Sciences Vocational School, Atatürk University, Erzurum 25030, Turkey; arzu-kavaz23@hotmail.com; 4 Science and Technology Application and Research Center, Aksaray University, Aksaray 68100, Turkey; emrah.dikici25@gmail.com

**Keywords:** acetylcholinesterase, antibacterial, antioxidant, *Lamiaceae* species, phenolic profiles

## Abstract

The *Lamiaceae* family are utilized as ornamental, medicinal, and food supplements throughout the world. The current study focuses on a comparative analysis of the phenolic compositions and bioactivities (including antioxidant, anticholinergic, and antibacterial activities) of ethanolic extracts derived from the aerial parts of the two species (*Lavandula stoechas* L. and *Thymus sipyleus* Boiss). The presence of phenolic compounds and phytochemicals in the plant extracts was identified using the LC-MS/MS technique. The LC-MS/MS analysis revealed that vanillic acid (125,596.66 µg/L) was the most abundant phytochemical in *L. stoechas*. Kaempferol (8550.52 µg/L) was the most abundant substance in *Thymus sipyleus*. The assessment of the antioxidant efficacy of the species extracts was conducted using the DPPH (2.2-diphenyl-1-picryl-hydrazyl-hydrate), ABTS (2.2′-azino-bis (3-ethylbenzothiazoline-6-sulfonic acid)), Fe^3+^–Fe^2+^ reducing, and CUPRAC (Cu^2+^–Cu^+^ reducing) assays. The anticholinergic activity of the samples was determined using the acetylcholinesterase (AChE) inhibition assay. The results of antioxidant activity were higher in the *T. sipyleus* than in the *L. stoechas* ethanol extracts. The extracts of *L. stoechas* exhibited radical scavenging activity ranging from 15 to 18%, while *T. sipyleus* had activity effects ranging from 34% to 38%. The AChE inhibition potential for *L. stoechas* and *T. sipyleus* extracts as IC_50_ values were 0.221 ± 0.01 mg/mL and 0.067 ± 0.02 mg/mL, respectively. The antibacterial effects of the ethanolic extracts of these species against pathogenic bacteria isolates were determined using the MIC (minimal inhibitory concentration) method. These findings indicated that the extracts from *L. stoechas* and *T. sipyleus* possess the potential to be natural antioxidants in the realm of food preservation. Additionally, their antioxidant, anticholinergic, and antimicrobial properties suggest potential therapeutic utility in the management of certain diseases.

## 1. Introduction

Many plant species belonging to the *Lamiaceae* family are very common. Furthermore, the *Lamiaceae* family stands out as one of the most abundant among flowering plants, known for its extensive array of species possessing medicinal attributes [1]. This taxonomically complex family has a wide geographical distribution, extending into Europe, Asia, North Africa, and the Canary Islands. Nevertheless, a preponderance of its species demonstrates a notably concentrated presence in the Mediterranean region [2,3]. The plant species are generally in the form of fragrant herbs and shrubs and are an economically important plant family [4,5]. They are characterized as a source of different metabolites, such as essential oils and flavonoids. The use of *Lamiaceae* in aromatherapy and antiseptics has been practiced since ancient times [6].

*Lavandula stoechas* (LS) is a precious perennial plant of the *Lamiaceae* family. It has a worldwide distribution with 39 species and more than 100 varieties, most of which are of Mediterranean origin [6,7,8]. Generally, LS is a plant with development potential in Europe, Africa, and Asia, especially in Southern Europe and North Africa, adjacent to the Mediterranean [7,9]. LS is used in folk medicine in the treatment of rheumatic diseases and inflammatory problems, with uses dating back to ancient times. *Lavandula* species were used for the treatment of kidney diseases and disorders of the digestive system in the Mediterranean region. Additionally, it is worth noting that the extract from *Lavandula* species exhibits a memory-enhancing effect. Its antioxidant attributes play a pivotal role in mitigating memory decline by offering protection against neurodegenerative processes. *Lavandula* species have also been subjected to evaluation within the domain of complementary and alternative medicine, specifically for their potential therapeutic efficacy in addressing conditions such as depression, diabetes, and headaches [9]. The species contains various bioactive components such as flavonoids, sterols, catechins, tannins, leuco-anthocyanins, coumarins, polyphenols, tannins, and triterpenes [10,11,12]. It has been determined that the essential oils of LS have antibacterial, antifungal, antidepressant, sedative, anticholinesterase, anti-inflammatory, antioxidant, and spasmolytic activities [13,14]. The extracts of LS have important antimicrobial effects on Gram-negative bacteria. The essential oils of this plant have showed antibacterial effects on *Enterobacter aerogenes*, *Escherichia coli*, *Staphylococcus aureus*, *Pseudomonas aeruginosa*, and *Salmonella epidermidis* [15]. The phenolic compounds of lavender flowers include hydroxybenzoic acids, hydroxycinnamic acids, and flavonoids [13,14]. The essential oils of *Lavandula* are used in the perfumery, cosmetic, and pharmaceutical industries and in the treatment of burns, skin injuries, and wounds [16].

The spontaneous members of the *Thymus* genus exhibit considerable taxonomic diversity within the *Lamiaceae* family, which includes over 215 species with a global distribution. *Thymus* sp. represents one of the particularly significant subspecies [17,18]. The above-ground part of this plant group has important properties, such as preventing stomach and intestinal disorders, antiseptic, scar correcting, diuretic, anti-diarrheal, and antiviral effects. Products derived from the thymus are widely used in medicine, veterinary medicine, the manufacture of cosmetic products, and as additives to foods [1,19,20]. The extracts obtained from wild thyme are used in the pharmaceutical and medical industries as antimicrobial substances and in the food industry as flavoring agents [21]. Studies have shown that thyme extracts exhibit broad antibacterial activity by inhibiting the growth of both Gram-positive and Gram-negative bacteria [22]. It has been determined that ethanol extracts of thyme have strong inhibitory activity, especially against *Staphylococcus aureus*, *Bacillus*, *Shigella*, and *Escherichia coli* [23]. The leaf components of *Thymus* plants are endowed with a spectrum of bioactive attributes, including antimicrobial, antioxidant, anti-inflammatory, antiviral, and expectorant properties. These beneficial effects are attributable to the presence of compounds such as thymol, carvacrol, p-cymene, and α-terpinene found in their essential oils [24,25].

Antioxidants serve as protective agents for living organisms against oxidative damage inflicted by free radicals. Recent studies have focused on the development of powerful antioxidant compounds, with the aim of reducing the harmful effects of free radicals that arise as a result of cellular processes such as lipid peroxidation and nucleotide mutations in DNA in damaged human tissues [26,27,28,29]. The plant species (*Lamiaceae* family) possess compounds that are rich in these antioxidant substances and their antioxidant properties are due to their phenolic composition, consisting of phytochemicals such as phenolic acids and flavonoids [9,30,31,32].

In the literature, studies on the phenolic components contained in plant extracts and their bioactivities (antioxidant, antibacterial, and anticholinergic) have been carried out using various solvents. It has been suggested that ethanol is more effective than many solvents, especially water, for the extraction of phenolic compounds, and has it more antioxidant activity [14,18,32]. There are few studies evaluating the phenolic profiles of ethanol extracts of TS and LS species and their contribution to their bioactivities.

This study involved the assessment of phytochemical profiles and the determination of the antioxidant and antibacterial activities of LS and *Thymus sipyleus* (TS) through standard techniques: LC-MS/MS analysis, DPPH, Ferric Reducing Power, ABTS, and CUPRAC assays. Additionally, the anticholinergic activity of the samples was evaluated via AChE inhibition. It is noteworthy that no prior research has been conducted to compare and analyze the significant bioactive properties of LS and TS collected from the Aksaray region.

## 2. Results and Discussion

### 2.1. The Phenolic Compound Profiles of LS and TS

Phenolic compounds are secondary metabolites that generally contribute to the bioactivity of plant extracts [33]. Secondary metabolites are generally used in various ways in food, medicine, chemistry, cosmetics, and agriculture. These compounds can inhibit various types of cancer and exert a protective effect against cardiovascular disease [34]. The utilization of LC-MS/MS for the analysis of phytochemical compositions in plant-derived extracts enables the most sensitive and precise identification and quantification of phytochemical and phenolic compounds [35].

In general, there are many factors that affect the profiles of detected phenolic compounds, such as the extraction method of the samples, the extraction procedure, the structure of the plant material, the phytochemicals, the environment, and the nature of the phenolic compounds [36,37]. 

Phenolic compound profiles in ethanol extracts of LS and TS species were determined using validated methods used in previous studies [27,37]. LC-MS/MS parameters and calibration equations for standard phenolic compounds are presented in Table S1. The chromatogram of standards and phenolic extracts for both species are presented in Figures S1–S3. When the data on the degradation products of phenolic in both species are analysed together with the standards, it can be seen that the standard MRM values given in Table 1 and the degradation products of phenolic compounds in both of the species overlap. The determination values (R^2^) of the standard curves were determined to be in the range of 0.99–1.00.

The limit of detection (LOD) values were in the range of 0.101–206.6 μg/L, while the limit of quantification (LOQ) varied in the range of 0.333μg/mL–214.3 μg/L. Please see Table 1. Vanillic acid was the most abundant component (125,596.66 µg/L) in LS. In a study conducted to investigate the phytochemistry of *Lavandula stoechas*, the identification and quantification of phenolic compounds and flavonoids in the methanol extract were carried out through high-performance liquid chromatography/mass spectrometry (HPLC-TOF/MS). Rosmarinic acid (80.89%) was found to be the most important constituent, followed by 4-hydroxybenzoic acid, caffeic acid, gentisic acid, ferulic acid, p-coumaric acid, vanillic acid, protocatechuic acid, and salicylic acid [38]. Since many of the phenolic compounds in the methanol extracts mentioned in the study were also detected in our study, the results support each other. The phenolic contents of the ethanol and methanol extracts were found to be higher than those of water extracts [1]. In this study, luteolin was detected in both LS and TS ethanol extracts. This luteolin was previously detected in methanol and ethanol/water extracts of the above-ground components of *Lavandula viridis*, as well as in other *Lavandula* species, but not in the water extract [14]. In another study, many phenolic compounds such as luteolin, caffeic acid, chlorogenic acid, gallic acid, vanillic acid, kaempferol, and p-hydroxybenzoic acid isolated from *Thymus* species (*T. vulgaris*, *T. capitatus*, *T. quinquecostatus*, etc.) were reported. In this study, it was also emphasized that bioactivities such as antioxidant and antimicrobial activities were attributed to the richness of phenolic content [18].

Luteolin (8550.52 µg/L) was the most abundant component in TS. The number and amounts of compounds determined in TS were higher than those in LS, with the exceptions of quercetin and vanillic acid. In another study, phenolic monoterpenes were found to be the main components of *Thymus* species. It was stated that thymol and carvacrol were the dominant compounds among the phenolics in the content. It was also emphasized that they have antimicrobial and antioxidant properties due to this content [39]. In our study, the antioxidant and antimicrobial activity in TS and LS can be attributed to the phenolic compound contents in these species.

### 2.2. Antioxidant Properties of LS and TS

Free radicals can cause irreversible cellular changes by altering proteins, lipids, and nucleic acids, which accelerate the aging process and cause many diseases such as Alzheimer’s, Parkinson’s, cancer, diabetes, and cardiovascular diseases [40]. The antioxidant capacity of phenolic compounds is due to their free radical scavenging ability, binding metal ions, and donating hydrogen atom electrons [41]. In general, phenolic and flavonoid compounds prevent the formation of chronic diseases, heart diseases, Alzheimer’s, cancer, diabetes, and other metabolic syndromes [42,43].

Free radicals may show activity as oxidizable substrates and can be reduced by different methods [44]. DPPH analysis is a method used to reveal the extent of oxidative cell damage that may occur in components such as polyunsaturated fatty acids, DNA, lipoproteins, and proteins in the organism [29]. Free radical capture values of ethanol extracts of LS and TS were determined by using two different methods; these are reported in Table 2.

The radical scavenging potential values of TS (38.854 ± 4.024%; 34.77 ± 2.88%) for the DPPH and ABTS assays were higher than those of the LS species (15.86 ± 1.30%; 18.75 ± 1.60%, respectively) at the concentration of 0.3 mg/ mL (Table 2). DPPH and ABTS radical scavenging activities of ethanol extracts of these two species were found to be lower than the standards, and these values were in the range of 71–83% for BHA and 46–49% for BHT. A study evaluated the phytochemical content analysis and antioxidant activity of *Lavandula stoechas* (collected from Antalya) methanol extract. The extract showed DPPH radical scavenging activity of 84.45 ± 5.1% at 1 mg/mL. This activity was attributed to phenolics such as quercetin, rutin, rosmarinic acid, and caffeic acid, which were more abundant in the content [35]. In this study, the DPPH radical scavenging potential of the ethanol extract of TS collected in Aksaray was found to be higher than that in most of the *Thymus* species in the Balkan Peninsula mentioned in the literature [3]. Although the antioxidant scavenging activity of the two plant extracts was lower than the standard compounds, TS ethanol extract showed higher activity than the LS extracts. From the obtained results, it can be stated that the radical scavenging activities of the plants may be attributed to the phenolic compounds present in the content, primarily including caffeic acid and kaempferol, which are abundant in TS.

The CUPRAC detection method is a fast, stable, simple, selective, and effective antioxidant detection method for a wide variety of polyphenolic substances [45]. The antioxidant activity of a compound is attributable to the fact that it provides hydroperoxide decomposition and prevents the use of transition metals that can catalyze Fenton-type reactions [46]. When evaluating the metal reduction activity of LS and TS ethanol extracts, although the Fe^3+^–Fe^2+^ reduction activity is lower than the standards, the extracts exhibited metal reduction activities that are close to the standards according to the CUPRAC assay (Table 2). In line with these results, it can be said that some phenolic compounds in LS and TS show significant radical scavenging and metal reduction capacity. As in the radical scavenging activity results, the metal reduction capacity of TS ethanol extracts was found to be higher than that of LS. An important positive correlation was determined between the DPPH, ABTS, FRAP (ABTS/FRAP methods, *p* < 0.05, r = 0.8932), and CUPRAC values and total flavonoid presence [47,48]. Similarly, Mrkonjić et al. (2021) reported that wild thyme plant ethanolic extracts had high antioxidant activity in ABTS, DPPH, and FRAP analyses [49].

Caffeic acid (CA) is a plant metabolite known for its high antioxidant potential. CA exhibits a higher antioxidant activity (H_2_O_2_ capture; capturing capacity of the ABTS^•+^/DPPH^•^) than standard compounds such as ascorbic acid and trolox [50]. In this study, TS is predicted to have greater radical scavenging and metal reduction potential due to its richer CA content.

### 2.3. Anticholinergic Effect of LS and TS

Compounds with anticholinergic effects are widely evaluated and used in treating Alzheimer’s disease (AD). The acceleration in the enzymatic hydrolysis by AChE of the neurotransmitter acetylcholine (ACh) is associated with the onset of AD. This disease, which affects brain tissue, causes significant destruction of brain tissues due to ACh deficiency, together with behavioral and memory impairment, especially among elderly people [51,52]. AChE inhibitors are extensively employed in the management of AD, elevating the production of antioxidants and shield cells against oxidative harm [53,54]. Observing Table 2, the LS and TS ethanol extracts had an inhibition property on the AChE at the level of IC_50_: 0.221 ± 0.01 μg/mL and 0.067 ± 0.02 μg/mL, respectively. There are many important studies on the use of AChE inhibitors in the treatment of AD due to the increase in acetylcholine levels in the brain tissue [26,50,52,55]. A group of researchers revealed in their study that the components of the aqueous extract of LS slowed down or prevented dementia [56]. It was emphasized in the previous study that the in vitro AChE inhibitor activity of ethanol extracts obtained from the wild plant *Lavandula viridis* was higher than water/ethanol and water extracts [14]. This study supports the purported benefits of LS in preventing memory-related disorders. Knowing the effects of *Lamiaceae* family species and their phenolic compounds on the improvement of neurodegenerative diseases may provide an approach that may contribute to the treatment of AD due to their AChE inhibition effects.

### 2.4. Antibacterial Effects of LS and TS

The antibacterial effects of LS and TS plants against *Staphylococcus aureus* ATCC 12600 (S.a.), *Escherichia coli* ATCC 11775 (E.c.), *Pseudomonas aeruginosa* ATCC 27853 (P.a.), and *Klebsiella pneumonia* ATCC 13883 (K.p.) tester strains are shown in Table 3. The extract of LS had the highest inhibition effect on *S. aureus*, but the lowest inhibition effect on *P. aeruginosa*. On the other hand, TS’s extract exhibited moderate inhibitory effects on *S. aureus* and *K. pneumonia*. The obtained results revealed that the plant extracts generally acted on the investigated microorganisms, but various concentrations of TS extract were more effective than LS. The results indicate that the two observed plant extracts had strong antibacterial effects on the tested pathogenic bacteria with different MIC values. However, the antibacterial effect of both plant extracts on the bacterial cultures studied was lower than the antibiotic neomycin. Similarly, Li et al. [18] reported that some phenolic components, especially luteolin, caffeic acid, chlorogenic acid, gallic acid, vanillic acid, kaempferol, and p-hydroxybenzoic acid found in *Thymus* species had strong antioxidant and antimicrobial activities due to the richness of phenolic content.

The obtained results revealed that the plant extracts generally had inhibitory effects on the investigated microorganisms. The LS extract was found to have an MIC value of 80 mg/mL against some bacterial strains except *Acinetobacter baumanii* and *P. aeruginosa* [57]. Our study results revealed that the extracts of these two species can be used for medicinal purposes, especially against the pathogenic microorganisms tested here. In another report, *T. serpyllum* had high antimicrobial activity against *S. aureus* and *C. Tropicalis*, and they showed low effects on *S. Enteritidis*, *P. aeruginosa* [58], *E. coli*, *Salmonella* sp., and *S. aureus* [59]. The extract of wild thyme was also previously reported to have antibacterial effects against *Listeria monocytogenes*, *Enterococcus faecalis*, *Bacillus cereus*, *E. coli*. *S. aureus*, *Yersinia enterocolitica*, and *Salmonella enterica* [60].

## 3. Materials and Methods

### 3.1. Chemicals

In the LC–MS/MS analysis, the following compounds were used as standards: Alizarin (97%), acetohydroxamic acid (98%), butein (≥97.0%), caffeic acid (98%), catechin hydrate (99%), ellagic acid (≥96.0%), fumaric acid (99%), gallic acid (97.5%), quercetin (95%), oleuropein (98%), myricetin (96%), kaempferol (97%), luteolin (99%), naringenin (98%), phloridzin dehydrate (99%), rutin hydrate (94%), resveratrol (99%), salicylic acid (≥99.0%), 4-Hydroxybenzoic acid (99%), and vanillic acid (≥97.0%). These were purchased from Sigma Aldrich (Darmstadt, Germany). Stock solutions (1 mg/mL) were prepared from each phenolic standard in MeOH. Working solutions were prepared at 1 mg/L by taking appropriate portions from the stock solutions. The compounds used for the study (1,1-diphenyl-2-picryl-hydrazyl (DPPH), butylated hydroxyanisole (BHA), butylated hydroxytoluene (BHT), 2,2′-azino-bis(3-ethylbenzthiazoline-6-sulfonic acid) (ABTS), α-tocopherol, ethanol, and HPLC-grade methanol) were obtained from Sigma (Sigma Aldrich, Darmstadt, Germany).

### 3.2. Plant Material

These wild-growing plant species were collected when they reached maturity from arid and steppe areas in and around Aksaray, where plant diversity and richness are high. *Lavandula stoechas*, referred to as LS here, and *Thymus sipyleus*, referred to as TS here, were collected as the fresh aerial parts of the plants from their natural habitat. These plants were collected from Aksaray, Turkey (Latitude: 37.88. Longitude: 33.35, Altitude: 980 m), at harvest time: LS in May, 2021, and TS in July, 2021. The samples were oven-dried at 40 °C until a constant weight was achieved. The dried materials were stored in jars in a moisture-free environment for the analyses. Voucher specimens were prepared and logged at the university herbarium for identification.

### 3.3. Preparation of Extracts

The shrub parts of the dried plant (LS and TS) stems were separated, and the remaining parts were used for the study. The aerial parts (upper part of stems and their leaves) were ground, and the plant material (10 g) was extracted with 70% aqueous ethanol (1:10 *w*/*v*) for 24 h on a magnetic stirrer. Afterwards, it was filtered through a filter paper (Whatman No. 1, Sigma Aldrich, Germany) to remove insoluble substances from the mixture. Then, the solvent was removed with the help of a rotary evaporator under reduced pressure. The resulting extract was dried under a stream of cold air and stored at −20 °C until use [61]. The yield of the ethanol extract obtained from the plant is in the range of 10–20% (*w*/*w*). Ethanol extracts of LS and TS were prepared at varying concentrations to perform all tests.

### 3.4. Phenolic Compound Analysis by LC-MS/MS

A liquid chromatograph–mass spectrometer (LC-MS/MS, Shimadzu, Kyoto, Japan) was used to analyze 20 distinct phenolic compounds. After the optimization study for 20 high-purity-standard phenolic compounds, the phenolic compounds in the plant extracts were quantitatively analysed. Phenolic compound profiles of plants species were determined using validated methods used in previous studies. The phenolic compound determination in LS and TS species was performed using the developed method by Yilmaz [27,37]. The system was coupled to an 8030 triple quadruple detector (Shimadzu, Kyoto, Japan) controlled by LabSolution Ver.5.6 software. Phenolic detection of the ethanolic extracts of LS and TS were conducted utilizing LC-MS/MS, employing a Nexera model Shimadzu HPLC coupled with a tandem MS device. The analytical setup included a CTO-10ASVP column furnace, DGU-20A3R degasser, LC-30AD dual pumps, SIL-30AC autosampler, and a C18 Inertsil ODS-4 column (3.0 mm × 100 mm, 2 µM) purchased from Shimadzu, Kyoto, Japan. The elution gradient comprised mobile phase A (0.1% formic acid and water) and mobile phase B (0.1% formic acid and methanol). Parameters for the instrument were set with a 4 µL injection volume and a solvent flow rate of 0.5 mL/min [37,61,62].

The gradient elution program started with 20% B for 0 min, increasing linearly from 50% for 8 min, then to 95% B at 12 min. Finally, the system returned to its original condition over 0.1 min and equilibrated for 3 min (total run time 15 min). Multiple reaction monitoring processing (MRM) mode was used to quantify the analysis. Two applications were made for each compound analysis in the experiment. The first was performed for the quantitative results and the second was analyzed for confirmation. The optimized MS parameters for phenolic compounds are given in Table 1 and Figure S1.

### 3.5. Ferric Reducing Power Assay

The iron-reducing capacities of samples were found using the modified method of Oyaizu (1986). The samples of increasing concentration from the stock solutions prepared at 1 mg/mL were added to the glass tubes with the help of a micropipette. The final volume was adjusted to 1 mL using distilled water. Subsequently, 2.5 mL of phosphate buffer (0.2 M. pH 6.6) and 2.5 mL of 1% potassium ferricyanide were introduced into these test samples within the tubes, followed by thorough vortexing. They were incubated at 50 °C for 15–20 min. It was centrifuged at 3000 rpm for 10 min by adding 2.5 mL of 10% TCA and 0.25 mL of 0.1% FeCl_3_. The absorbances of the mixtures were read at 700 nm to determine the iron-reducing capacity. Results were compared with standard reference compounds [63,64].

### 3.6. Cu^2+^ Reducing Assay

The copper ion reduction capacity of the total components of the ethanol extract was determined by using a slightly modified version of the method used in previous studies [45,65]. The samples of increasing concentration from the stock solutions prepared at 1 mg/mL were added to the glass tubes with the help of a micropipette. A measure of 1 mL of acetate buffer (1.0 M), neocuproin (7.5 mM) as the chromogenic reducing agent, and CuCl_2_ solution (10 mM) were added to each tube and vortexed. The tubes were made up to 1 mL with distilled water and incubated at 25 °C for 30 min. The absorbances of the mixtures were read at 450 nm to determine the reducing capacity. The results were compared with the standard reference compounds.

### 3.7. DPPH Radical Scavenging Assay

The DPPH free radical scavenging capacity of the total compounds in the ethanol extract was determined using the Blois method [66,67]. First, stock 0.1 mM DPPH solution was prepared. Afterward, the samples whose concentration increased from the stock solutions (ethanol extract and standard references) prepared at 1 mg/mL were pipetted into glass tubes with the help of a micropipette, and their final volume was made up to 2 mL with 96% ethanol. Then, 0.5 mL of the prepared DPPH radical solution was added to the tubes containing the sample and standard references and incubated in the dark for 30 min by vortexing. The decreasing absorbances of the mixtures were read at 517 nm to determine the radical removal capacity. Results were compared with standard reference compounds.

### 3.8. ABTS Radical Scavenging Assay

The ABTS free radical scavenging capacity of the total compounds in the ethanol extract was determined according to the method developed by Re et al. (1999) [68]. First of all, ABTS cation radicals were produced from 7 mM ABTS solution by adding 2.45 nM persulfate to the solution. The absorbance of the solution was adjusted to 0.750 ± 0.050 at 734 nm by diluting the buffer solution (pH 7.4. 0.1 M). Afterwards, the samples whose concentration increased from the stock solutions (ethanol extract and standard references) prepared at 1 mg/mL were pipetted into glass tubes with the help of a micropipette, and their final volume was made up to 2 mL with 96% ethanol. Then, 0.5 mL of the prepared ABTS cation radicals solution was added to the tubes containing the sample and standard references and incubated in the dark for 30 min by vortexing. The decreasing absorbances of the mixtures were read at 734 nm to determine the radical removal capacity. Results were compared with standard reference compounds.

### 3.9. Anticholinergic Assay

The inhibitory potential of LS and TS ethanol extract against AChE was determined according to the modified Ellman method [43,50,69]. A measure of 100 µL of Tris/HCl buffer (pH 8.0. 1 M)., 50 µL of 5.5′-dithiobis (2-nitrobenzoic acid) (DTNB) (0.5 mM), 20 µL of AChE solution (2.5 × 10^−3^ EU), and 20–150 µL of LS and TS ethanol extract solutions at 1 mg/mL were mixed. The reaction was then initiated by the addition of acetylthiocholine iodide (50 µL), which was used as the substrate. Finally, the varying absorbances of the mixtures were read at 412 nm to determine the inhibitory potential. The change in AChE activity due to increasing concentrations of the samples was evaluated. The IC_50_ values were calculated from activity (%) versus different concentrations for samples of ethanol extracts.

### 3.10. Antibacterial Activity Using the Minimum Inhibitory Concentration (MIC) Method

The minimum inhibitory concentration (MIC) of the ethanolic LS and TS extracts for antibacterial activity was determined using the micro-dilution bioassay in 96-well micro-plates. The 100 μL of each resuspended sample were two-fold serially diluted with sterile distilled water down a 96-well microplate for each of the four bacteria in duplicate. A similar two-fold serial dilution of neomycin (Sigma Aldrich, Darmstadt, Germany) (0.1 mg/mL) was used as a positive control against each bacterium. Water and bacteria-free broth were included as negative controls. Overnight cultures (incubated at 37 °C in a water bath with shaking) of four bacterial strains—*Staphylococcus aureus* ATCC 12600 (S.a.)*, Escherichia coli* ATCC 11775 (E.c.), *Pseudomonas aeruginosa* ATCC 27853 (P.a.), and *Klebsiella pneumonia* ATCC 13883 (K.p.) bacteria—were diluted with sterile Mueller–Hinton (MH) broth (1 mL bacteria/50 mL MH). Then, 100 μL of each bacterial culture was added to each well. The plates were covered with parafilm and incubated overnight at 37 °C. Bacterial growth was tested by adding 50 µL of 0.2 mg/mL *p*-iodonitrotetrazolium chloride (INT) to each well and the plates were incubated at 37 °C for 1 h. Bacterial growth in the wells was indicated by a red–pink color, whereas clear wells indicated inhibition of growth by the tested sample. MIC values were recorded as the lowest concentration of extract, showing a clear well. Each assay was repeated twice [32,70,71].

### 3.11. Statistical Analysis

Statistical analyses of the data were evaluated by unpaired Student’s *t*-test using GraphPad Prism version 8 (GraphPad Software, La Jolla, CA, USA). All results were given as means with their standard deviation (SD). *p* < 0.05 was taken as the minimum level of significance.

## 4. Conclusions

In this study, LS and TS ethanol extracts were compared in terms of phenolic compounds, metal reduction, free radical scavenging activities, and anticholinergic and antibacterial properties. It was determined that the extracts of these species had significant effects in terms of the specified parameters. The fact that bioactivities such as metal reduction, radical scavenging, and anticholinergic activities in the TS extract were found to be higher than those in the LS extract. It can be attributed to the higher levels of luteolin, resveratrol, caffeic acid, fumaric acid, gallic acid, vanillic acid, and kaempferol in the TS extract. The data obtained in this study revealed that LS and TS ethanol extracts can be used in the food, pharmaceutical, and cosmetic industries as a food supplement and a source of natural antioxidants. In addition, the measurements obtained from this study revealed that the extracts of these two species could be used as a source of bioactive molecules and antibacterial agents. Also, the findings showed that these two species were rich sources of antioxidants and natural AChE inhibitors. These species can be used for the treatment of AD due to their AChE inhibition effects.

## Figures and Tables

**Table 1:** Phenolic compound concentration of LS and TS extracts.

Standard Compounds	^a^ MRM	^b^ RSD %	^c^ LOD/LOQ (μg/L)	Recovery (%)	^d^ RT (LS/TS)	LS (µg/L)	TS (µg/L)
Quercetin	301.10 > 151.00	0.0136	22.5/25.7	1.001	3.947/3.885	180.34	104.93
Acetohydroxamic acid	76.10 > 58.00	0.0082	2.8/8.2	1.000	0.356/0.407	53.15	108.36
Catechin hydrate	291.10 > 139.00	0.0236	8.2/11.4	0.994	-/2.721	N.D.	4.51
Vanillic acid	168.80 > 93.00	0.0062	125.5/142.2	1.001	3.128/3.528	125,596.66	2690.72
Resveratrol	229.10 > 135.00	0.0131	9.0/13.6	0.998	3.183/3.201	75.66	431.64
Fumaric acid	115.20 > 71.00	0.0047	25.2/31.3	0.997	0.508/0.514	125.37	400.48
Gallic acid	-	-	-	-	-	N.D.	N.D.
Caffeic acid	179.20 > 135.00	0.0137	6.3/10.7	1.009	2.751/2.776	159.36	2504.97
Phloridzin dihydrate	435.00 > 273.10	0.0564	61.0/207.0	1.000	3.441/3.195	26.96	39.37
Oleuropein	539.10 > 377.20	0.0694	0.05/1.0	0.997	-/3.582	N.D.	34.07
Ellagic acid	-	-	-	-	-	N.D.	N.D.
Myricetin	-	-	-	-	-	N.D.	N.D.
Protocatechuic acid	-	-	-	-	-	N.D.	N.D.
Butein	271.10 > 135.00	0.0145	22.7/28.6	0.096	3.849/3.857	40.07	147.49
Naringenin	271.10 > 150.90	0.0205	5.4/6.4	0.998	3.865/3.875	369.60	750.43
Luteolin	285.20 > 150.90	0.0057	0.5/2.5	1.007	4.349/3.988	4481.48	8550.52
Kaempferol	285.10 > 116.90	0.0144	206.6/214.3	0.999	4.349/3.987	354.44	154.28
Alizarin	239.20 > 211.00	0.0351	65.2/77.5	0.966	4.594/4.834	5.55	35.55
4-Hydroxybenzoic acid	137.20 > 93.00	0.0154	30.5/40.25	0.996	3.510/3.528	78.60	30.87
Salicylic acid	137.20 > 93.00	0.0124	4.2/7.6	1.009	3.512/3.528	88.56	39.29

^a^ MRM: multiple reaction monitoring. ^b^ RSD %: relative standard deviation. ^c^ LOD/LOQ (µg/L): limit of detection/limit of quantitation. ^d^ RT: retention time. N.D: not detected.

**Table 2:** The AChE inhibitory potential and antioxidant activity of LS and TS.

Antioxidants	DPPH ^a^ (0.3 mg/mL)	ABTS ^a^ (0.3 mg/mL)	FRAP Assay ^b^ (0.2 mg/mL)	CUPRAC Assay ^b^ (0.2 mg/mL)	AChE	
					IC_50_ (mg/mL)	R^2^
LS	15.86 ± 1.30	18.75 ± 1.60	0.18 ± 0.02	0.42 ± 0.05	0.221 ± 0.01	0.953 ± 0.02
TS	38.85 ± 4.02	34.77 ± 2.88	0.25 ± 0.01	0.68 ± 0.04	0.067 ± 0.02	
BHA	71.64 ± 6.17	82.95 ± 6.37	0.43 ± 0.06	0.61 ± 0.04		
BHT	46.67 ± 3.41	48.79 ± 3.20	0.65 ± 0.08	0.66 ± 0.05		
Trolox	82.63 ± 6.37	79.68 ± 5.31	0.28 ± 0.01	0.52 ± 0.04		
Galanthamine					1.6 ± 0.06 (µM)	

Standards (BHA—butylated hydroxyanisole; BHT—butylated hydroxytoluene; Trolox; galanthamine). R^2^—determination coefficient; LS—*Lavandula stoechas*; TS—*Thymus sipyleus.* ^a^ Values are expressed as percent radical scavenging activity in ethanol extracts (0.3 mg/mL). ^b^ Values are expressed as absorbance. High absorbance indicates high metal reduction capacity in ethanol extracts (0.2 mg/mL).

**Table 3:** Antibacterial effects (MIC) of ethanlic extracts of LS and TS.

Plant Extract	Bacterial MIC (mg/mL)			
	S.a.	E.c.	P.a.	K.p.
LS	0.78	5.15	12.5	1.56
TS	1.56	6.25	6.25	2.50
Neomycin (positive control)	1.6 × 10^−3^	0.8 × 10^−3^		0.8 × 10^−3^

S.a.—*Staphylococcus aureus*. E.c.—*Escherichia coli*. P.a.—*Pseudomonas aeruginosa*. K.p.—*Klebsiella pneumonia*. LS—*Lavandula stoechas*; TS—*Thymus sipyleus*.

## Data Availability

Data are contained within the article and supplementary materials.

## Supplementary Materials

The following supporting information can be downloaded at: https://www.mdpi.com/article/10.3390/molecules29020480/s1, Table S1. LC-MS/MS parameters and calibration equations for standard phenolic compounds. Figure S1. Representative LC-MS/MS chromatogram for standard phenolic compounds. Figure S2. The fragmentation pattern from MS analyses for phenolics in the LS extract. Figure S3. The fragmentation pattern from MS analyses for phenolics in the TS extract.
