# Supplementary material for: Phenolic Content Analysis of Two Species Belonging to the Lamiaceae Family: Antioxidant, Anticholinergic, and Antibacterial Activities
Source: Molecules. 2024 Jan 18;29(2):480. doi: 10.3390/molecules29020480 (PMC10821218; doi:10.3390/molecules29020480)
Supplement: Supplementary file 1 [file molecules-29-00480-s001.zip › molecules-2782656-supplementary.pdf]

Table S1. LC-MS/MS parameters and calibration equations for standard phenolic compounds.

Standard compounds	ESI ION MOD	[a]MRM	[b]RSD %	[C]LOD/LOQ (μg/L)	Recovery (%)	[d]RT	Equation
Quercetin	neg	301.10>150.90	0.0136	22.5/25.7	1.001	3.891	Y=(13.7831)X+(-146.951)
Acetohydroxamic acid	pos	76.15>58.00	0.0082	2.8/8.2	1.000	0.406	Y = (150.982)X + (23.1833)
Catechin hydrate	neg	291.00>139.10	0.0236	8.2/11.4	0.994	2.532	Y = (79.2933)X + (-2406.22)
Vanillic acid	pos	168.95>65.00	0.0062	125.5/142.2	1.001	2.762	Y = (48.0522)X + (-876.904)
Resveratrol	pos	229.00>135.00	0.0131	9.0/13.6	0.998	3.606	Y = (46.4361)X + (-1314.61)
Fumaric acid	neg	115.20>71.10	0.0047	25.2/31.3	0.997	0.809	Y = (20.2986)X + (-762.592)
Gallic acid	neg	169.10>124.90	0.0136	0.90/1.6	1.000	1.278	Y = (65.3835)X + (-2699.84)
Caffeic acid	neg	179.00>135.00	0.0137	6.3/10.7	1.009	2.836	Y = (124.785)X + (-487.132)
Phloridzin dihydrate	neg	435.10>273.10	0.0564	61.0/207.0	1.000	3.594	Y = (33.4069)X + (-1396.90)
Oleuropein	neg	539.10>377.00	0.0694	0.05/1.0	0.997	3.567	Y = (25.9240)X + (-558.916)
Ellagic acid	neg	301.10>228.90	0.0856	0.101/0.333	1.002	3.681	Y = (13.1516)X + (717.421)
Myricetin	neg	317.00>179.10	0.0079	55.4/59.6	0.999	3.644	Y = (5.25903)X + (-1167.31)
Protocatechuic acid	neg	181.00>108.00	0.0129	30.3/35.4	1.011	3.556	Y = (37.0934)X + (2684.23)
Butein	neg	271.00>134.90	0.0145	22.7/28.6	0.096	3.935	Y = (526.954)X + (23026.1)
Naringenin	neg	271.00>150.90	0.0205	5.4/6.4	0.998	3.952	Y = (49.3543)X + (367.917)
Luteolin	neg	285.00>150.90	0.0057	0.5/2.5	1.007	4.069	Y = (317.241)X + (33733.3)
Kaempferol	neg	285.00>117.00	0.0144	206.6/214.3	0.999	4.298	Y = (34.6668)X + (3721.79)
Alizarin	neg	239.00>211.00	0.0351	65.2/77.5	0.966	4.594	Y = (2.63905)X + (-206.494)
4-Hydroxybenzoic acid	neg	137.20>93.10	0.0154	30.5/40.25	0.996	3.555	Y = (3.97487)X + (1614.23)
Salicylic acid	neg	137.20>93.00	0.0124	4.2/7.6	1.009	3.558	Y = (735.804)X + (-498.102)

[a]MRM: Multiple Reaction Monitoring. [b]RSD %: Relative standard deviation. [c]LOD/LOQ (μg/L): Limit of detection/ limit of quantitation. [d]RT: Retention time. [e]R2: Determination coefficient. N.D: Not detected.

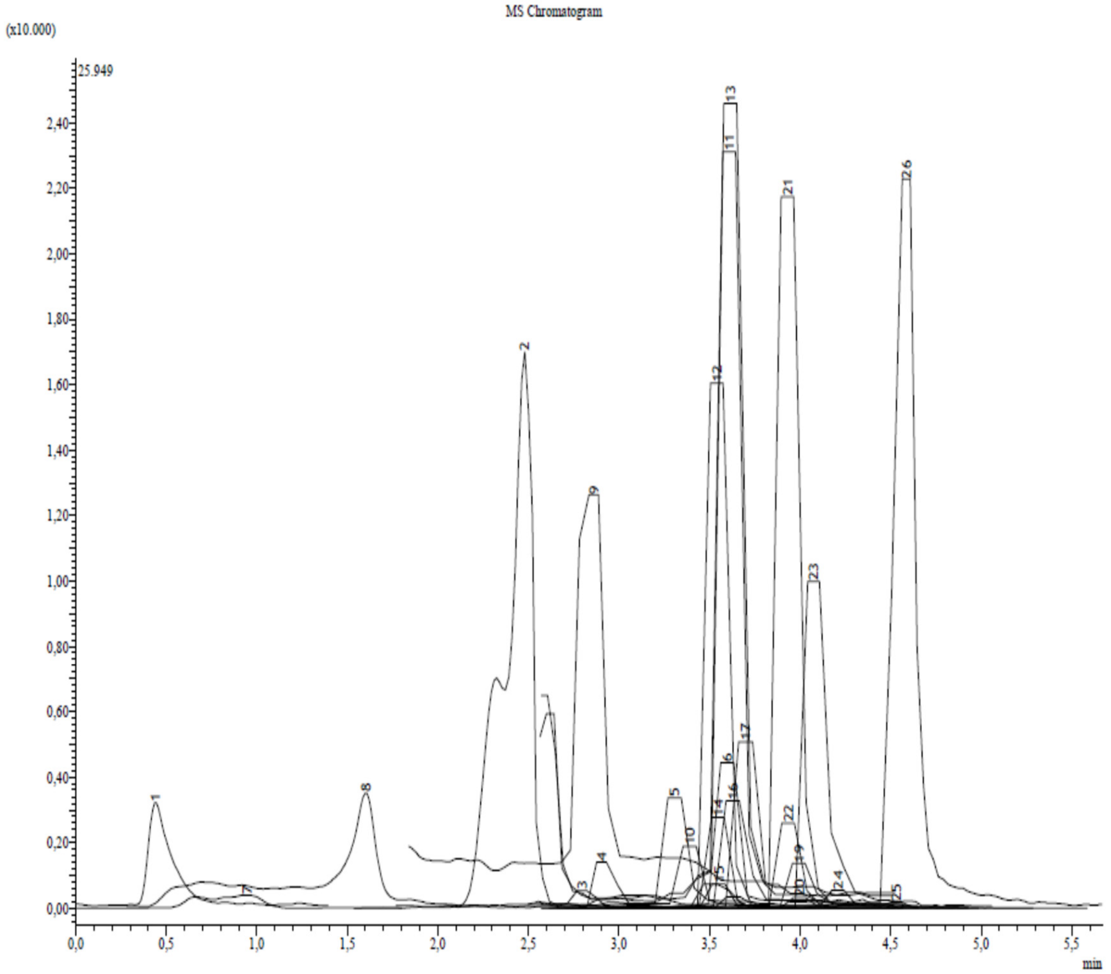

Figure S1. Representative LC-MS/MS chromatogram for standard phenolic compounds.

	Name
1	Acetohydroxamic Acide_poz
2	Catechinhyrate_pos
3	Vanilic Acide_poz
4	Syringic acid_pos
5	Thymoquinone_poz
6	Resveratrol_pos
7	Fumaric Acid _neg
8	Gallic acid_neg
9	Caffeic acid_neg
10	Hydoxycinamic_neg*
11	4-Hydroxybenzoic acid _neg
12	Protocatechuic acid _neg
13	Salicylic acid_neg

14	Oleuropein_neg
15	Phloridzindyhrate_neg
16	2-hyroxy1.4nph_neg
17	Myricetin_neg
18	Ellagic acid_neg
19	Quercetin_neg
20	Bütein_neg
21	Naringenin_neg
22	Silymarin_neg
23	luteolin_neg
24	Kaempferol_neg
25	Alizarin_neg
26	Curmin_neg

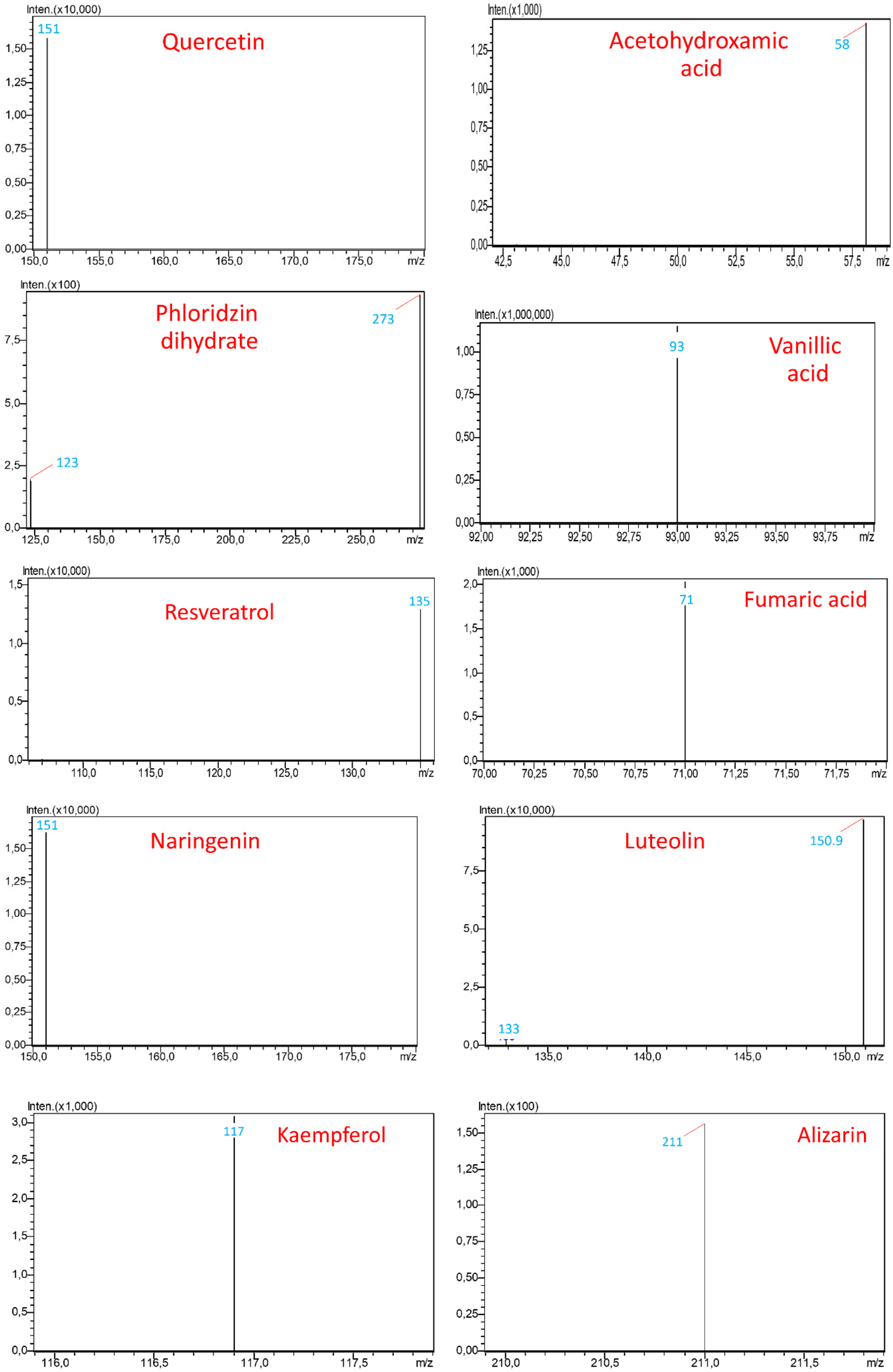

Inten.(x10,000)
151
Quercetin
m/z
Inten.(x1,000)
Acetohydroxamic acid
58
m/z
Inten.(x100)
Phloridzin dihydrate
273
123
m/z
Inten.(x1,000,000)
93
Vanillic acid
m/z
Inten.(x10,000)
Resveratrol
135
m/z
Inten.(x1,000)
71
Fumaric acid
m/z
Inten.(x10,000)
151
Naringenin
m/z
Inten.(x10,000)
Luteolin
150.9
133
m/z
Inten.(x1,000)
117
Kaempferol
m/z
Inten.(x100)
211
Alizarin
m/z

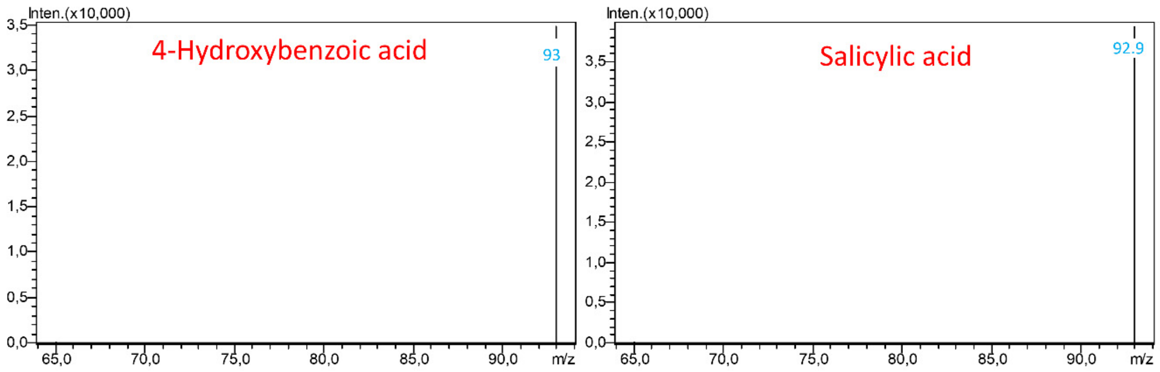

Figure S2. The fragmentation pattern from MS analyses for phenolics in the LS extract

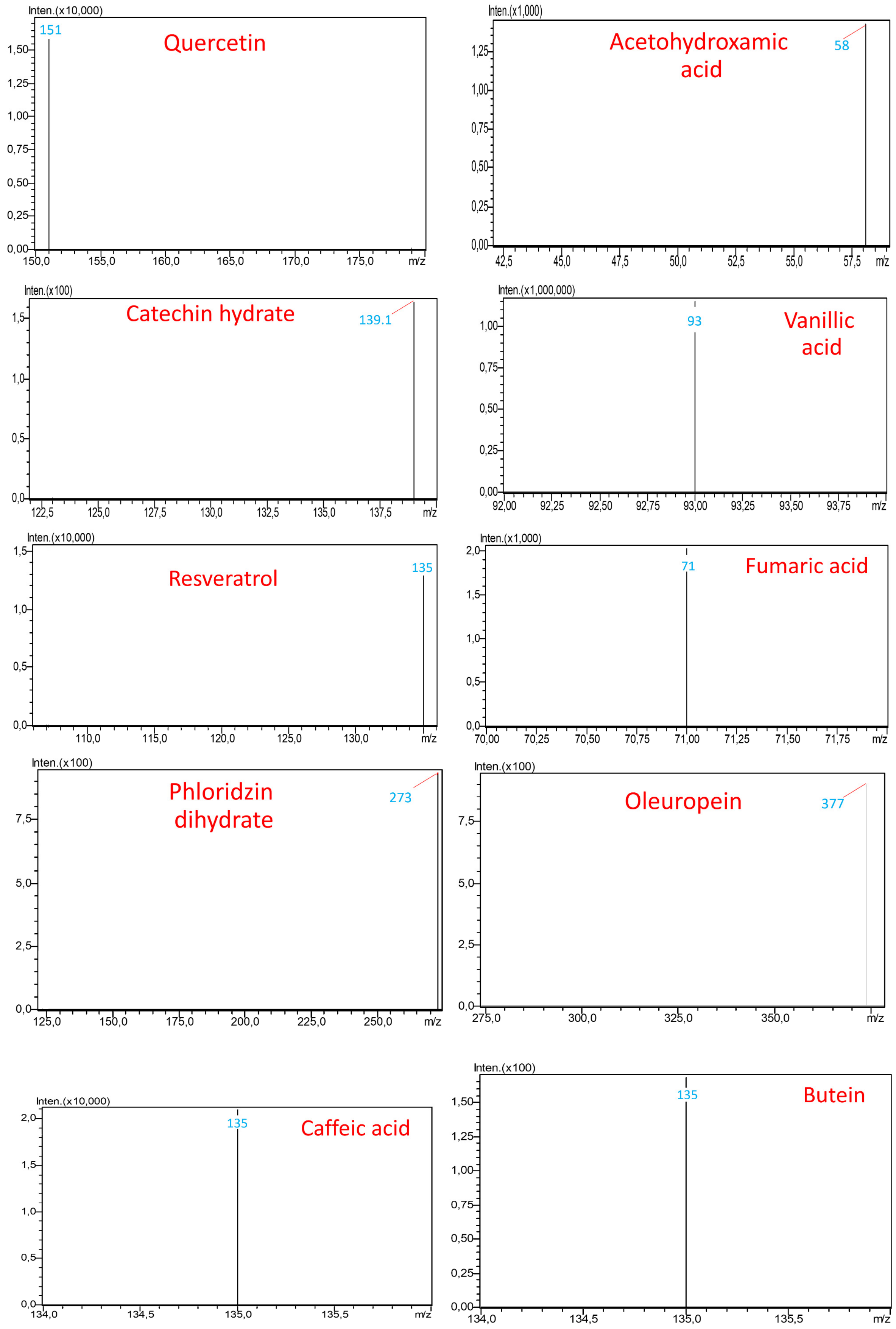
Inten.(x10,000)
151
Quercetin
m/z
Inten.(x1,000)
Acetohydroxamic acid
58
m/z
Inten.(x100)
Catechin hydrate
139.1
m/z
Inten.(x1,000,000)
93
Vanillic acid
m/z
Inten.(x10,000)
Resveratrol
135
m/z
Inten.(x1,000)
71
Fumaric acid
m/z
Inten.(x100)
Phloridzin dihydrate
273
m/z
Inten.(x100)
Oleuropein
377
m/z
Inten.(x10,000)
135
Caffeic acid
m/z
Inten.(x100)
135
Butein
m/z

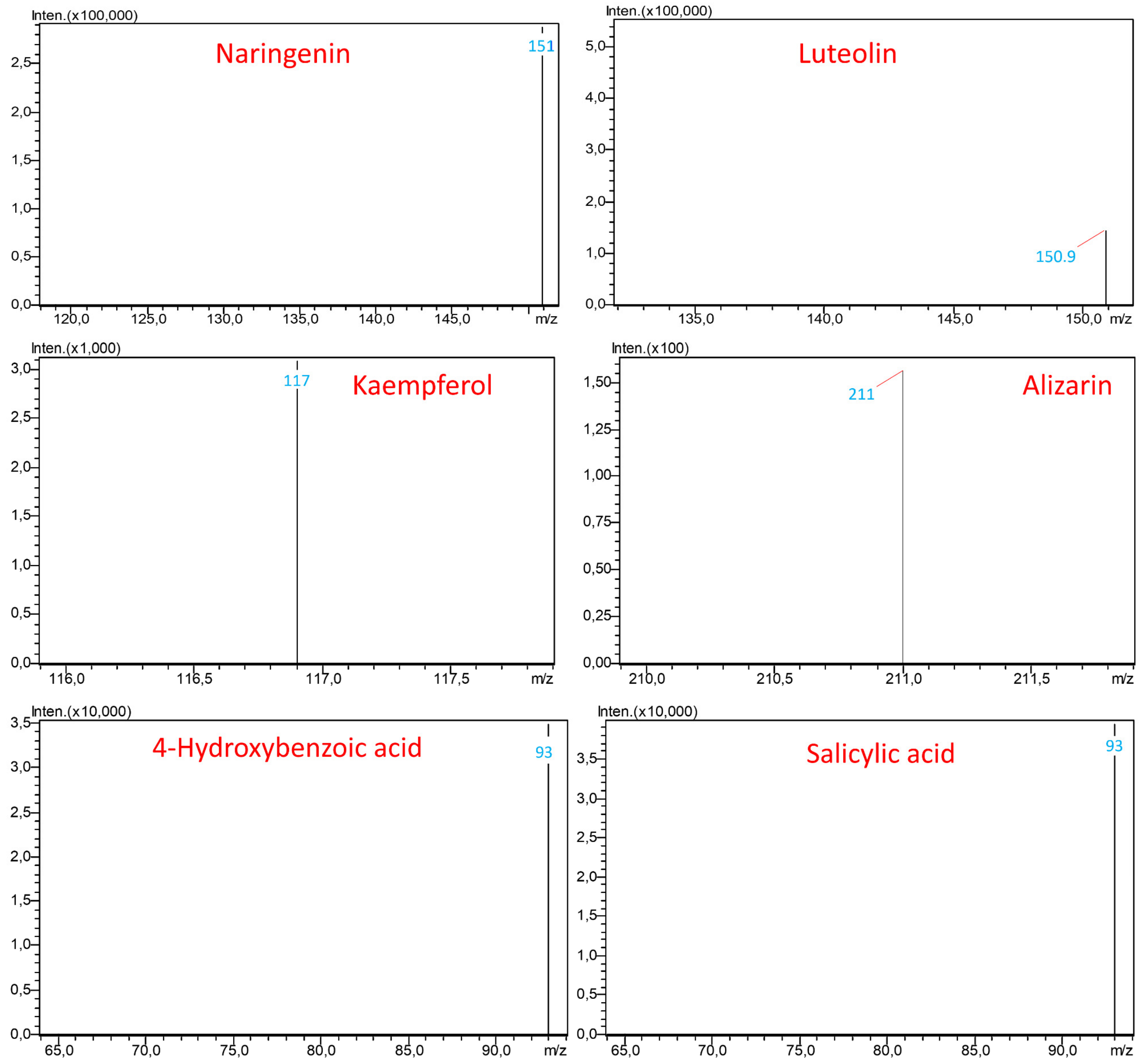

Figure S3. The fragmentation pattern from MS analyses for phenolics in the TS extract
